# Assessment of Hospital Overcrowding Perceptions and Outpatient Care Quality Among Hypertensive Patients in Mampong, Ghana: A Cross‐Sectional Study

**DOI:** 10.1002/hsr2.72322

**Published:** 2026-04-08

**Authors:** Godfred Darko, Courage Aguadze, Patience Dabuo

**Affiliations:** ^1^ Department of Public Health Education, Faculty of Environment and Health Education Akenten Appiah‐Menka University of Skills Training and Entrepreneurial Development Kumasi Ghana; ^2^ Department of Nursing, Faculty of Nursing and Midwifery University for Development Studies Tamale Ghana

**Keywords:** Ghana, hypertension, perceived hospital overcrowding, perceived outpatient care quality

## Abstract

**Background and Aims:**

Hospital overcrowding is a common challenge in low‐ and middle‐income countries and tends to reduce the quality of outpatient care for patients with chronic conditions such as hypertension. Evidence in municipal health settings in Ghana is limited. This study assessed the association between patients' perceptions of hospital overcrowding and their perceived outpatient care quality among hypertensive patients in Mampong Ashanti Municipality, Ghana.

**Methods:**

An analytical cross‐sectional study was conducted among 342 hypertensive outpatients at Mampong Municipal Hospital and Kofiase Health Centre (October–December 2025). Participants were selected using systematic random sampling with proportional allocation. Data were collected using structured questionnaires assessing perceived hospital overcrowding and outpatient service quality (adapted SERVQUAL). Descriptive statistics summarized participant characteristics, Chi‐square tests examined associations, and multivariable logistic regression identified predictors of perceived outpatient care quality.

**Results:**

A total of 61.7% of respondents reported moderate to high perceived overcrowding. Participants were predominantly ≥ 60 years (52.1%), female (62.6%), with comorbidities (58.5%). Higher perceived overcrowding was associated with poorer outpatient care quality. Each one‐unit increase in perceived overcrowding score was associated with 20.8% lower odds of reporting good outpatient care quality (AOR = 0.79; 95% CI: 0.75–0.84). Patients aged 40–49 years (AOR = 9.32; 95% CI: 2.57–33.88; *p* < 0.001) and 50–59 years (AOR = 3.02; 95% CI: 1.04–8.82; *p* = 0.04) were more likely to report good care quality compared with those ≥ 70 years. Lower education attainment, income (< GHS 1000), lack of health insurance, and single marital status were associated with lower odds of perceived outpatient care quality.

**Conclusion:**

Perceived hospital overcrowding is associated with lower perception of outpatient care quality among hypertensive patients. Interventions to decongest outpatient departments and address socioeconomic disparities are essential to improve hypertension care.

## 1 Introduction

Hypertension affects over 1.28 billion adults worldwide, contributing substantially to cardiovascular morbidity and mortality [1]. Most care occurs in outpatient settings, where regular follow‐up, medication review, and patient education are critical [2]. However, health systems globally face increasing pressure from rising chronic disease prevalence [3]. Hospital outpatient departments often operate beyond capacity, leading to overcrowding [4]. Studies from high‐ and low‐income countries show that overcrowded clinics are associated with long waiting times, shortened consultations, and reduced patient satisfaction [5]. These conditions limit comprehensive clinical assessment, disrupt continuity of care, and reduce opportunities for counseling. For chronic conditions like hypertension, these service constraints may contribute to poorer disease control and long‐term outcomes [6].

Overcrowding affects care through multiple pathways. High patient volume reduces consultation time and increases provider workload [7]. Clinicians often prioritize speed over thoroughness, leaving little room for detailed medication review or lifestyle counseling [8]. Communication with patients suffers, and follow‐up planning is inconsistent. Prolonged overcrowding also contributes to provider fatigue and burnout, further undermining service quality [9]. Evidence indicates that these factors increase missed appointments, reduce adherence, and delay therapeutic adjustments [10]. In hypertensive patients, such disruptions translate into poor blood pressure control and increased risk of complications. Thus, overcrowding functions as a systemic barrier to effective outpatient care and sustained chronic disease management [11].

In Africa, non‐communicable diseases now account for approximately 37% of all deaths, with hypertension as the most prevalent cardiovascular risk factor [12]. Health systems face persistent staff shortages, limited infrastructure, and weak referral pathways [13]. In Sub‐Saharan Africa, patients frequently bypass primary care and attend hospitals for routine follow‐up, intensifying congestion in outpatient departments [14]. Studies report long waiting times and high patient loads per clinician, limiting comprehensive assessment, patient education, and follow‐up scheduling [15]. More than 50% of hypertensive patients remain uncontrolled, partly due to these systemic challenges [16]. Overcrowding thus amplifies inequities in chronic disease management and contributes to preventable complications [17].

Ghana reflects these broader patterns. National estimates place adult hypertension prevalence at approximately 30.3% [18], with hypertension among the leading causes of outpatient morbidity [19]. Outpatient departments in public hospitals frequently experience high attendance with constrained staff and limited clinic space [20]. In the Ashanti Region of Ghana, outpatient visits accounted for 5.4% of total attendance at local health facilities, reflecting the demand for routine healthcare services [21]. Even with the National Health Insurance Scheme, many patients face substantial indirect costs related to time spent seeking care [22]. Nearly half of hypertensive patients in Ghana experience catastrophic health expenditure, driven mainly by transport and productivity loss [23], and over 50% of treated patients have uncontrolled blood pressure [24].

Despite evidence of overcrowding in many Ghanaian hospitals, few studies have examined how it relates to outpatient care experiences among hypertensive patients in municipal settings. Most research has focused on prevalence, treatment adherence, and financial burden, with limited attention to service quality from the patient's perspective. For individuals managing hypertension, outpatient clinics are central to ongoing care, and crowded environments may shape how patients perceive the quality of services received. This study therefore assessed the association between patients' perceptions of hospital overcrowding and their perceived outpatient care quality in Mampong, Ashanti Region, Ghana.

## 2 Methodology

### 2.1 Study Design

This study employed an analytical cross‐sectional design to assess the association between patients' perceptions of hospital overcrowding and their perceived outpatient care quality among hypertensive patients in Mampong, Ashanti Region, Ghana. This design was appropriate because it allowed for the simultaneous measurement of the exposure (perceived hospital overcrowding) and the outcome (perceived outpatient care quality) and the examination of their relationship at a single point in time. Data collection was conducted from October 8 to December 16, 2025.

### 2.2 Study Area

The study was conducted in Mampong Municipality, located in the Ashanti Region of Ghana. The municipality was purposively selected due to its rapidly growing population, driven by expanding educational institutions, administrative offices, and commercial activity, which have collectively increased demand for healthcare services [25].

The municipality's public health facilities, including Mampong Municipal Hospital and Kofiase Health Centre, serve as primary points of care for chronic conditions such as hypertension. High outpatient attendance at these facilities often results in overcrowded waiting areas, long consultation times, and strained healthcare resources [26]. These conditions make Mampong an ideal setting to examine the association between perceived hospital overcrowding and patients' experiences of outpatient care quality [27].

### 2.3 Study Population and Eligibility Criteria

The study population comprised adult patients diagnosed with hypertension who were receiving outpatient care at Mampong Municipal Hospital and Kofiase Health Center, both located within the Mampong Ashanti Municipal, Ghana. Participants were recruited from the outpatient departments of the two facilities during routine hypertension clinic visits. These public health facilities provide primary and secondary healthcare services to residents within the municipality and surrounding communities and manage a high volume of patients with chronic conditions, including hypertension.

Eligible participants were adults aged 30 years and above with a confirmed diagnosis of hypertension who were attending outpatient clinics at either facility during the study period. Participants were required to have attended the outpatient clinic at least once prior to the study to ensure sufficient exposure to the outpatient care environment and its service delivery conditions. Only patients who were clinically stable, able to understand the study procedures, and willing to provide informed consent were included.

Patients were excluded if they were admitted as inpatients or receiving emergency care at the time of data collection, as their care experiences differ from routine outpatient services. Newly diagnosed hypertensive patients attending the clinic for the first time were also excluded due to limited exposure to outpatient care processes. Additionally, patients with severe illness, cognitive impairment, and communication difficulties that could compromise their ability to provide reliable responses were excluded from the study [28].

### 2.4 Sample Size Determination and Sampling Technique

The sample size for this study was determined using Yamane's formula for finite populations at a 5% margin of error [29]. Yamane's formula is given as: $n=\frac{N}{{1+N(e)}^{2}}$, where *n* is the sample size, *N* is the population size, and *e* is the margin of error. The total outpatient hypertensive population at the study facilities was 2330, comprising 1825 patients at Mampong Municipal Hospital and 505 patients at Kofiase Health Centre. Applying Yamane's formula yielded a sample size of 342 participants. The sample was proportionally allocated to reflect the distribution of patients across the two facilities, with 267 participants from Mampong Municipal Hospital and 75 participants from Kofiase Health Centre. Proportional allocation ensured that the sample accurately represented the outpatient hypertensive population.

Participants were selected using a systematic random sampling technique. The outpatient attendance registers of each facility served as the sampling frame. The sampling interval (*n*th value) was calculated by dividing the facility population by the proportional sample size, resulting in an interval of 7 for both Mampong Municipal Hospital and Kofiase Health Centre. A random starting point between 1 and 7 was chosen at each facility, and every seventh patient on the register was selected until the required sample size was achieved [30]. This method ensured equal opportunity for all eligible patients to be included while maintaining proportional representation. This improved the reliability and external validity of the study results.

### 2.5 Data Collection Instruments and Variables

Data were collected using structured self‐administered questionnaires designed to capture patient perceptions of hospital overcrowding and outpatient service quality. Two instruments were employed: the Patient‐Perceived Hospital Overcrowding Questionnaire and the Patient‐Perceived Outpatient Service Quality Questionnaire (SERVQUAL–Healthcare Adapted). The complete Hospital Overcrowding and SERVQUAL questionnaires are provided as Supporting File 1. Both instruments were developed based on a review of the literature and adapted to reflect the local healthcare context of this study. Additionally, these instruments captured patients' self‐reported perceptions, and no objective metrics of hospital crowding or outpatient service quality (e.g., measured waiting times or patient‐to‐staff ratios) were collected.

The sociodemographic and clinical variables included age, sex, marital status, educational level, monthly income, residence, health insurance status, duration of hypertension, and presence of comorbidities. These were treated as covariates to explore associations with perceived hospital overcrowding and outpatient service quality.

The Perceived Hospital Overcrowding Questionnaire contained 10 items assessing patient perceptions of crowding in waiting areas, staff availability, consultation delays, administrative processes, and the overall impact of overcrowding on care [31]. Items were scored on a 5‐point Likert scale (1 = strongly disagree, 5 = strongly agree), with higher scores indicating greater perceived overcrowding. The reliability of this instrument was assessed during pretesting at Jamasi Health Center, a primary healthcare facility serving a population similar to the study sites, among 40 hypertensive patients. The pretest yielded a Cronbach's alpha of 0.897, which showed excellent internal consistency prior to the main study.

The Perceived Outpatient Service Quality Questionnaire (SERVQUAL) consisted of 25 items grouped into five domains: tangibles, reliability, responsiveness, assurance, and empathy. Items were scored on a 5‐point Likert scale (1 = strongly disagree, 5 = strongly agree), with higher scores reflecting better perceived outpatient service quality [32]. Pretesting of the SERVQUAL instrument among the 40 patients demonstrated strong reliability across domains: tangibles (*α* = 0.872), reliability (*α* = 0.858), responsiveness (*α* = 0.854), assurance (*α* = 0.861), and empathy (*α* = 0.804). The overall Cronbach's alpha for all 25 items was 0.959. While these instruments demonstrated excellent internal consistency, the reliance on self‐reported data introduces the possibility of Common Method Variance (CMV) and response bias, which may affect the observed associations.

### 2.6 Data Analysis

Data were checked for completeness, coded, and entered into Microsoft Excel and SPSS version 26.0. Descriptive statistics (frequencies and percentages) summarized participants' sociodemographic and clinical characteristics and their distribution across levels of outpatient service quality. All 342 completed questionnaires (100%) were included in the analysis, as there were no missing or incomplete responses. The study was conducted and reported in accordance with the STROBE guidelines, and statistical analyses followed SAMPL recommendations for transparent reporting. Chi‐square tests assessed associations between categories of perceived hospital overcrowding, perceived outpatient service quality and participants' sociodemographic and clinical characteristics.

The main analysis employed binary logistic regression. Overall perceived outpatient service quality scores, measured using the SERVQUAL instrument, were dichotomized at the sample median (50th percentile). Scores above the median were classified as “higher perceived service quality,” while scores at or below the median were classified as “lower perceived service quality.” The median was selected due to the absence of a validated threshold for this population, enabling relative comparison within the study sample. Although analyzing the outcome as a continuous variable using linear regression was considered, logistic regression was preferred to facilitate interpretation of odds ratios and to align with the categorical nature of commonly used service quality benchmarks [33]. However, we acknowledge that dichotomization of a continuous variable may reduce statistical power and variability.

To assess the robustness of the findings and address potential bias from dichotomizing the SERVQUAL score, a sensitivity analysis using linear regression was conducted, treating outpatient service quality as a continuous outcome. The results were consistent with the logistic regression model, showing that higher perceived hospital overcrowding was associated with lower perceived outpatient care quality (*β* = −1.49, *p* < 0.001). These results confirm that the observed association was not an artifact of dichotomization. Full regression coefficients and model diagnostics are provided in Supporting File 2 (Supporting Table 1). Multicollinearity was evaluated using Variance Inflation Factors (VIF) and tolerance statistics derived from an auxiliary linear regression model, including all independent variables. All VIF values were below 2.1 and tolerance values exceeded 0.48, indicating no evidence of multicollinearity.

Although Table 2 presents hospital overcrowding as low, moderate, and high for descriptive purposes, the logistic regression treated the underlying 10‐item scale as a continuous predictor, with the AOR reflecting a one‐unit increase in perceived overcrowding. Sociodemographic and clinical variables were included as covariates based on prior empirical and theoretical evidence, and were specified a priori. All primary analyses were pre‐specified prior to data analysis; no post hoc subgroup analyses were conducted. All variables were self‐reported, introducing potential common method variance and response bias. Model fit was assessed using the Hosmer–Lemeshow goodness‐of‐fit test. Results are presented as odds ratios (ORs) with 95% confidence intervals (CIs). All tests were two‐sided, with statistical significance set at *p* < 0.05.

### 2.7 Ethical Consideration

Approval for the study was granted by the Ghana Health Service Ethics Review Committee, referenced as (GHS‐ERC: 053/09/25). The research adhered to the principles outlined in the Declaration of Helsinki for studies involving human participants [34]. All participants were cognitively able to provide informed consent. Participants received detailed explanations regarding the objective of the study, the methods to be used, and any possible risks. Participation was entirely voluntary, and respondents were informed that they could discontinue at any stage without any impact on their access to healthcare. Measures were taken to protect confidentiality, including the assignment of unique codes and secure storage of all collected data.

## 3 Results

### 3.1 Sociodemographic and Clinical Characteristics of Respondents

Table 1 shows the sociodemographic and clinical characteristics of the 342 respondents included in the analysis. Most participants were aged 60 years and above, with those aged 70 years or older constituting the largest proportion (27.5%), followed by respondents aged 60–69 years (24.6%). Females accounted for 62.6% of the study population, while males represented 37.4%.

**TABLE 1:** Sociodemographic and clinical characteristics of respondents.

Variable	Category	Frequency (*n*)	Percentage (%)
Age of respondents (years)	30–39	46	13.4
	40–49	51	14.9
	50–59	67	19.6
	60–69	84	24.6
	≥ 70	94	27.5
Sex	Male	128	37.4
	Female	214	62.6
Marital status	Single	38	11.1
	Married	137	40.1
	Divorced	63	18.4
	Widowed	104	30.4
Educational level	No formal education	121	35.4
	Basic/JHS	96	28.0
	Secondary	71	20.8
	Tertiary	54	15.8
Monthly income (GHS)	< 1000	141	41.2
	1000–1999	82	24.0
	2000–2999	61	17.8
	≥ 3000	58	17.0
Place of residence	Urban	175	51.2
	Suburban	59	17.2
	Rural	108	31.6
Health insurance status	No	138	40.4
	Yes	204	59.6
Duration of hypertension	< 5 years	224	65.5
	5–10 years	87	25.4
	> 10 years	31	9.1
Comorbidities	Yes	200	58.5
	No	142	41.5

Regarding marital status, married participants formed the largest group (40.1%), followed by widowed respondents (30.4%). Over one‐third of participants (35.4%) had no formal education, while 28.1% had basic education, 20.8% had secondary education, and 15.8% had attained tertiary education. Monthly income was predominantly low, with 41.2% earning less than 1000 Ghana cedis, and 24.0% earning between 1000 and 1999 Ghana cedis.

More than half of the respondents resided in urban areas (51.2%), while 31.6% lived in rural communities. Health insurance coverage was reported by 59.6% of participants. Most respondents had been diagnosed with hypertension for less than 5 years (65.5%), while 25.4% reported a duration of 5–10 years. Comorbid conditions were present among 58.5% of the participants.

### 3.2 Perceived Hospital Overcrowding Among Hypertensive Patients by Sociodemographic and Clinical Characteristics

Table 2 shows the distribution of perceived hospital overcrowding among hypertensive patients by sociodemographic and clinical characteristics. Overall, high perceived overcrowding was most frequently reported by participants aged ≥ 70 years (52.1%), females (46.7%), and those without formal education (57.0%). Patients with lower monthly income (< 1000 GHS, 60.3%), rural residents (34.3%), those without health insurance (30.4%), and participants with comorbidities (49.5%) also reported higher levels of perceived overcrowding. In contrast, duration of hypertension was not significantly associated with perceived overcrowding (*χ*² = 7.65, df = 4, *p* = 0.11). All other sociodemographic and clinical variables showed statistically significant associations with perceived hospital overcrowding (*p* < 0.05).

**TABLE 2:** Perceived hospital overcrowding among hypertensive patients by sociodemographic and clinical characteristics.

Variable	Category	Low *n* (%)	Moderate *n* (%)	High *n* (%)	*χ*² (df)	*p*‐value
Age (years)	30–39	14 (30.4)	22 (47.8)	10 (21.7)	19.41 (8)	0.01
	40–49	6 (11.8)	29 (56.9)	16 (31.4)		
	50–59	12 (17.9)	32 (47.8)	23 (34.3)		
	60–69	15 (17.9)	36 (42.9)	33 (39.3)		
	≥ 70	13 (13.8)	32 (34.0)	49 (52.1)		
Sex	Male	29 (22.7)	68 (53.1)	31 (24.2)	17.37 (2)	< 0.001
	Female	31 (14.5)	83 (38.8)	100 (46.7)		
Marital status	Single	10 (26.3)	14 (36.8)	14 (36.8)	20.97 (6)	0.002
	Married	26 (19.0)	74 (54.0)	37 (27.0)		
	Divorced	4 (6.3)	27 (42.9)	32 (50.8)		
	Widowed	20 (19.2)	36 (34.6)	48 (46.2)		
Educational level	No formal education	6 (5.0)	46 (38.0)	69 (57.0)	51.60 (6)	< 0.001
	Basic/JHS	15 (15.6)	43 (44.8)	38 (39.6)		
	Secondary	25 (35.2)	32 (45.1)	14 (19.7)		
	Tertiary	14 (25.9)	30 (55.6)	10 (18.5)		
Monthly income (GHS)	< 1000	9 (6.4)	47 (33.3)	85 (60.3)	71.66 (6)	< 0.001
	1000–1999	17 (20.7)	33 (40.2)	32 (39.0)		
	2000–2999	14 (23.0)	38 (62.3)	9 (14.8)		
	≥ 3000	20 (34.5)	33 (56.9)	5 (8.6)		
Place of residence	Urban	17 (9.7)	74 (42.3)	84 (48.0)	26.68 (4)	< 0.001
	Suburban	16 (27.1)	33 (55.9)	10 (16.9)		
	Rural	27 (25.0)	44 (40.7)	37 (34.3)		
Insurance	No	28 (20.3)	68 (49.3)	42 (30.4)	6.11 (2)	0.05
	Yes	32 (15.7)	83 (40.7)	89 (43.6)		
Duration of illness	< 5 years	45 (20.1)	98 (43.8)	81 (36.2)	7.65 (4)	0.11
	5–10 years	12 (13.8)	43 (49.4)	32 (36.8)		
	> 10 years	3 (9.7)	10 (32.3)	18 (58.1)		
Comorbidities	Yes	20 (10.0)	81 (40.5)	99 (49.5)	32.84 (2)	< 0.001
	No	40 (28.2)	70 (49.3)	32 (22.5)		

### 3.3 Perceived Outpatient Service Quality by Sociodemographic and Clinical Characteristics

Among the 342 hypertensive patients (Table 3), 38.3% reported dissatisfaction with perceived outpatient service quality. Dissatisfaction was highest among patients aged ≥ 70 years (58.5%) and lowest among those aged 40–49 years (17.6%). Female patients had higher dissatisfaction (46.3%) compared to males (25.0%). Educational level showed a clear gradient, with 54.5% of patients with no formal education dissatisfied vs. 16.7% of those with tertiary education. Dissatisfaction decreased with increasing monthly income, from 58.2% among patients earning < 1000 GHS to 10.3% among those earning ≥ 3000 GHS. Place of residence and health insurance status did not show significant differences. Longer duration of hypertension (> 10 years) was associated with higher dissatisfaction (61.3%), and patients with comorbidities reported more dissatisfaction (45.5%) than those without (28.2%). All associations were statistically significant (*p* < 0.05), except for place of residence and health insurance.

**TABLE 3:** Perceived outpatient service quality by sociodemographic and clinical characteristics.

Variable	Category	Dissatisfied *n* (%)	Satisfied *n* (%)	*χ*² (df)	*p*‐value
Age	30–39	10 (21.7)	36 (78.3)	36.847 (4)	< 0.001
	40–49	9 (17.6)	42 (82.4)		
	50–59	18 (26.9)	49 (73.1)		
	60–69	39 (46.4)	45 (53.6)		
	≥ 70	55 (58.5)	39 (41.5)		
Sex	Male	32 (25.0)	96 (75.0)	15.321 (1)	< 0.001
	Female	99 (46.3)	115 (53.7)		
Educational level	No formal education	66 (54.5)	55 (45.5)	33.857 (3)	< 0.001
	Basic/JHS	41 (42.7)	55 (57.3)		
	Secondary	15 (21.1)	56 (78.9)		
	Tertiary	9 (16.7)	45 (83.3)		
Monthly income (GHS)	< 1000	82 (58.2)	59 (41.8)	51.669 (3)	< 0.001
	1000–1999	31 (37.8)	51 (62.2)		
	2000–2999	12 (19.7)	49 (80.3)		
	≥ 3000	6 (10.3)	52 (89.7)		
Place of residence	Urban	74 (42.3)	101 (57.7)	2.406 (2)	0.30
	Suburban	20 (33.9)	39 (66.1)		
	Rural	37 (34.3)	71 (65.7)		
Health insurance	No	49 (35.5)	89 (64.5)	0.766 (1)	0.38
	Yes	82 (40.2)	122 (59.8)		
Duration of hypertension	< 5 years	80 (35.7)	144 (64.3)	7.652 (2)	0.02
	5–10 years	32 (36.8)	55 (63.2)		
	> 10 years	19 (61.3)	12 (38.7)		
Comorbidities	Yes	91 (45.5)	109 (54.5)	10.554 (1)	0.001
	No	40 (28.2)	102 (71.8)		

### 3.4 Association Between Patients' Perception of Hospital Overcrowding and Their Perceived Outpatient Care Quality

Results from Table 4 shows that higher perceived hospital overcrowding was associated with lower odds of reporting satisfactory outpatient service quality (AOR = 0.792; 95% CI: 0.750–0.836; *p* < 0.001). This indicates that each unit increase in perceived overcrowding was associated with a 20.8% reduction in the odds of patient satisfaction. Compared with patients aged ≥ 70 years, those aged 40–49 years (AOR = 9.324; 95% CI: 2.566–33.884; *p* < 0.001) and 50–59 years (AOR = 3.021; 95% CI: 1.035–8.815; *p* = 0.04) had higher odds of reporting satisfaction. Single participants were less likely to report satisfaction compared to widowed patients (AOR = 0.139; 95% CI: 0.036–0.536; *p* = 0.005). Similarly, respondents with no formal education (AOR = 0.177; 95% CI: 0.056–0.559; *p* = 0.005) and those with basic education (AOR = 0.181; 95% CI: 0.056–0.586; *p* = 0.006) had lower odds of satisfaction compared to those with tertiary education. Participants earning less than 1000 GHS monthly were less likely to report satisfaction compared to those earning ≥ 3000 GHS (AOR = 0.239; 95% CI: 0.065–0.879; *p* = 0.03). Lack of health insurance was also associated with lower odds of satisfaction (AOR = 0.467; 95% CI: 0.220–0.991*; p* = 0.05). Sex, place of residence, duration of hypertension, comorbidities, and secondary education were not significantly associated with outpatient care satisfaction. The logistic regression model demonstrated good fit (Hosmer–Lemeshow *χ*² = 8.28, df = 8, *p* = 0.406). Model explanatory indices were Cox & Snell *R*² = 0.471 and Nagelkerke *R*² = 0.639.

**TABLE 4:** Association between perceived hospital overcrowding and outpatient care quality among hypertensive patients: a multivariable logistic regression analysis.

Predictor variable	*β*	SE	Wald	*p*‐value	AOR (Exp(B))	95% CI for AOR
Perceived hospital overcrowding	−0.234	0.030	60.564	< 0.001*	0.792	0.750–0.836
Age (ref: ≥ 70 years)			17.807	0.001		
30–39 years	1.038	0.610	2.892	0.09	2.822	0.889–8.956
40–49 years	2.233	0.620	12.958	< 0.001*	9.324	2.566–33.884
50–59 years	1.105	0.545	4.121	0.04*	3.021	1.035–8.815
60–69 years	−0.137	0.505	0.074	0.79	0.872	0.341–2.229
Sex (ref: female)	−0.243	0.395	0.379	0.54	0.78	0.382–1.608
Marital status (ref: widowed)			9.383	0.03		
Single	−1.976	0.705	7.859	0.005*	0.139	0.036–0.536
Married	−0.063	0.465	0.018	0.89	0.939	0.391–2.254
Divorced	−0.328	0.522	0.394	0.53	0.721	0.274–1.894
Educational level (ref: tertiary)			8.954	0.03		
No formal education	−1.731	0.621	7.778	0.005*	0.177	0.056–0.559
Basic/JHS	−1.711	0.622	7.564	0.006*	0.181	0.056–0.586
Secondary	−1.092	0.690	2.503	0.11	0.336	0.091–1.238
Monthly income (GHS) (ref: ≥ 3000)			5.555	0.14		
< 1000	−1.432	0.645	4.928	0.03*	0.239	0.065–0.879
1000–1999	−0.837	0.664	1.591	0.21	0.433	0.125–1.498
2000–2999	−1.112	0.700	2.520	0.11	0.329	0.087–1.242
Place of residence (ref: rural)			4.980	0.08		
Urban	0.062	0.525	0.014	0.91	1.064	0.390–2.904
Sub‐urban	−1.048	0.571	3.364	0.07	0.351	0.118–1.040
Health insurance (ref: yes)	−0.762	0.379	4.035	0.05*	0.467	0.220–0.991
Duration of hypertension (ref: < 5 years)			2.910	0.23		
5–10 years	1.049	0.622	2.848	0.09	2.855	0.884–9.227
> 10 years	0.816	0.673	1.469	0.23	2.262	0.554–9.236
Comorbidities (ref: no)	0.814	0.523	2.424	0.12	2.256	0.791–6.428
Constant	8.718	1.431	37.139	< 0.001	6114.675	

*Note:* Model fit: (Hosmer–Lemeshow *χ*² = 8.282, df = 8, *p* = 0.406), with −2 Log Likelihood = 237.75, Cox & Snell *R*² = 0.471, and Nagelkerke *R*² = 0.639.

Abbreviations: *β*, regression coefficient; AOR, adjusted odds ratio; SE, standard error; CI, 95% confidence interval.

## 4 Discussion

This cross‐sectional study examined perceptions of hospital overcrowding and outpatient service quality among 342 hypertensive patients attending municipal facilities in Mampong, Ghana. Our findings indicate that many patients perceived outpatient departments as crowded, and these perceptions were associated with lower satisfaction with care. Patient experiences varied across age, education, income, marital status, and health insurance, highlighting how sociodemographic factors shape perceptions of service quality. Overall, nearly four in 10 patients reported lower satisfaction, with clear patterns emerging across different population subgroups.

### 4.1 Perceived Overcrowding and Care Experience

Patients' perceptions of hospital overcrowding varied across sociodemographic and clinical characteristics, with higher perceived crowding most commonly reported among older adults (52.1% ≥ 70 years), females (46.7%), low‐income earners (< 1000 GHS: 60.3%), individuals with no formal education (57.0%), and patients with comorbidities (49.5%). Urban residents reported the highest perceived overcrowding (48.0%) compared with suburban (16.9%) and rural (34.3%) participants. Duration of illness showed a trend toward higher overcrowding for those with longer disease histories (> 10 years: 58.1%). These perceptions likely reflect challenges in municipal outpatient services, such as long waiting times, limited staffing, and reduced opportunities for individualized attention [35]. Even moderate perceived crowding appears to influence how patients evaluate care, shaping their overall satisfaction and engagement with outpatient services. These findings are consistent with reports from other low‐ and middle‐income countries, where perceived congestion in public facilities is associated with longer waiting times, reduced patient–provider interaction, and diminished patient‐reported experiences of care [36].

### 4.2 Age‐Related Perceptions

Satisfaction differed across age groups. Older adults tended to perceive care less favorably, potentially due to mobility limitations, complex health needs, and the burdens of waiting in crowded outpatient departments [37]. Middle‐aged patients showed strikingly higher adjusted odds (AOR = 9.324 for 40–49 years), likely reflecting both substantive advantages, such as greater familiarity with healthcare systems, higher health literacy, and more flexible schedules, and statistical imprecision from smaller reference groups. These patterns are consistent with studies from South Africa and Nigeria, which suggest that age can influence patient expectations and satisfaction in outpatient settings [38, 39].

### 4.3 Socioeconomic Influences

Lower educational attainment, lower income, and lack of health insurance were linked to less favorable perceptions of outpatient care. Limited health literacy and financial constraints may reduce patients' confidence in navigating healthcare services [40], while out‐of‐pocket costs can heighten negative perceptions despite national insurance coverage [41]. These findings highlight the intersection between socioeconomic disadvantage and perceived service quality, a trend consistently observed in LMIC contexts [42].

### 4.4 Marital Status and Social Support

Single patients were more likely to report lower perceived service quality compared with those who were married (Table 4), suggesting that social support may buffer negative experiences [43]. Family or partner support may help patients manage clinic visits, reduce stress, and facilitate understanding of medical advice [44], which is particularly important for chronic conditions like hypertension where sustained engagement with care is critical [45].

### 4.5 Other Observations

While comorbidities, hypertension duration, and sex were not strongly associated with perceived care quality after adjustment, descriptive trends suggested that patients with longer disease histories or additional health conditions tended to perceive care less favorably [46]. These patterns indicate that more complex and sustained healthcare needs may interact with perceptions of overcrowding to shape overall experiences [47], even if the effects are modest. These robust patterns were supported by good multivariable model fit (Hosmer–Lemeshow *p* = 0.406).

### 4.6 Limitations and Strengths of the Study

This study had some limitations. It was conducted in only two public health facilities within the Mampong Municipality. The findings may therefore not represent outpatient experiences in other parts of Ghana. Data on hospital overcrowding and outpatient service quality were entirely based on patients' self‐reports, which may have been influenced by individual perceptions at the time of the interview. This reliance on subjective measures introduces the potential for Common Method Variance (CMV) and response bias, which may have affected the observed associations. No objective metrics of hospital crowding (e.g., patient‐to‐staff ratios or measured wait times) were collected, which limits the ability to validate patient perceptions.

Additionally, the cross‐sectional design captured information at a single point in time, preventing assessment of changes in perception of overcrowding or service quality over time. Furthermore, due to the simultaneous measurement of exposure and outcome, reverse causation cannot be ruled out; patients dissatisfied with service quality may have been more likely to perceive the hospital as overcrowded. Very ill patients, emergency cases, and newly diagnosed patients were excluded, so their experiences may be underrepresented. The odds ratios should be interpreted cautiously given the cross‐sectional design, reliance on self‐reported data, and possible response bias. The relatively high Nagelkerke R² (0.639) may partly reflect structural overlap between items in the adapted overcrowding questionnaire and the SERVQUAL instrument, as both tools assess similar domains such as waiting time and physical environment. This shared measurement variance may have artificially inflated the predictive power of the logistic regression model, and therefore the strength of the association should be interpreted with caution.

Despite these limitations, the study had important strengths. Participants had prior exposure to outpatient services, enhancing the reliability of their assessments. The inclusion of two healthcare facilities enhanced contextual relevance. Standardized tools were used for data collection. Multivariable logistic regression strengthened the analysis. Overall, the findings provide practical evidence to support outpatient service improvement and health system planning in similar settings.

## 5 Conclusion

This study found that higher levels of perceived hospital overcrowding were associated with lower patient‐reported satisfaction and perceived quality of care among hypertensive patients in the Ashanti Mampong Municipality. The findings suggest that managing hypertension effectively requires attention not only to clinical treatment but also to systemic and structural factors that may influence patients' experiences of care.

Economic and sociodemographic factors, particularly lower income, lack of health insurance, and lower educational attainment, were linked to poorer perceptions of service quality. Age and marital status disparities were also observed, with older and widowed patients reporting lower satisfaction, potentially reflecting challenges in accessing resources and support networks.

Overall, the results indicate that patients' experiences of outpatient care are shaped by a combination of perceived hospital overcrowding, socioeconomic vulnerability, and demographic differences. Interventions to improve patient satisfaction and perceived quality of care should therefore consider both operational improvements and broader systemic factors alongside standard medical care.

## 6 Implications for Policy and Practice

Perceived hospital overcrowding may affect patients' experiences of outpatient care. To mitigate this, healthcare facilities could implement strategies such as appointment staggering, triage prioritization, and dedicated hypertension clinics to optimize patient flow and reduce congestion. Regular monitoring of patient volume and staff allocation may also enhance service delivery.

Capacity building for healthcare providers in patient‐centered communication and care can improve patient experience. Infrastructure improvements, including additional consultation rooms and support staff, may further strengthen outpatient chronic care services. Community‐based hypertension programs could help reduce hospital visits while maintaining continuity of care.

Expanding health insurance coverage to include longer consultations and follow‐ups could improve access and equity, particularly for socioeconomically vulnerable patients. Policymakers should address both operational and systemic factors to promote timely, equitable, and satisfactory outpatient care for hypertensive patients.

## Author Contributions

**Godfred Darko:** conceptualization, data collection, data analysis, draft of initial manuscript, writing of final manuscript. **Courage Aguadze:** conceptualization, data analysis, writing of final manuscript. **Patience Dabuo:** data collection, draft of initial manuscript, writing of final manuscript. All authors read and approved the final version of the manuscript.

## Funding

The authors have nothing to report.

## Disclosure

The lead author Godfred Darko affirms that this manuscript is an honest, accurate, and transparent account of the study being reported; that no important aspects of the study have been omitted; and that any discrepancies from the study as planned (and, if relevant, registered) have been explained.

## Ethics Statement

Ethical approval was obtained from the Ghana Health Service Ethics Review Committee (GHS‐ERC: 053/09/25) prior to data collection. The study adhered to the principles of the Declaration of Helsinki and its subsequent amendments.

## Consent

Informed consent was obtained from all participants prior to their involvement in the study. Participants received comprehensive information regarding the study objectives, procedures, potential risks, and benefits. Participation was entirely voluntary, and individuals were informed of their right to withdraw at any time without any consequences.

## Conflicts of Interest

The authors declare no conflicts of interest.

## Supporting information


**Supporting File 1:** hsr272322‐sup‐0001‐Supplementary_File_1_Instruments

**Supporting File 2:** hsr272322‐sup‐0002‐Supplementary_File_2_Sensitivity_analysis

## Data Availability

The data that support the findings of this study are available from the corresponding author upon reasonable request.
