# Supplementary material for: Assessment of Hospital Overcrowding Perceptions and Outpatient Care Quality Among Hypertensive Patients in Mampong, Ghana: A Cross‐Sectional Study
Source: Health Sci Rep. 2026 Apr 8;9(4):e72322. doi: 10.1002/hsr2.72322 (PMC13062499; doi:10.1002/hsr2.72322)
Supplement: Supplementary file 1 — Supporting File 1: hsr272322‐sup‐0001‐Supplementary_File_1_Instruments [file HSR2-9-e72322-s002.docx]

**Assessment of Hospital Overcrowding Perceptions and Outpatient Care Quality among Hypertensive Patients in Mampong, Ghana: A Cross-Sectional Study**

**Instruments for Data Collection**

**Section A: Sociodemographic Information**

1. **Age (years):**
   - 30-39
   - 40-49
   - 50-59
   - 60-69
   - ≥70
2. **Sex:**
   - Male
   - Female
3. **Marital Status:**
   - Single
   - Married
   - Divorced
   - Widowed
4. **Educational Level:**
   - No formal
   - Basic/JHS
   - Secondary
   - Tertiary / Higher
5. **Monthly Income (GHS):**
   - <1000
   - 1000-1999
   - 2000-2999
   - ≥3000
6. **Residence:**
   - Urban
   - Suburban
   - Rural
7. **Health Insurance:**
   - No
   - Yes
8. **Duration of Illness:**
   - <5 years
   - 5–10 years
   - >10+ years
9. **Comorbidities:**
   - Yes
   - No

**Patient-Perceived Hospital Overcrowding Questionnaire**

**Instructions:** For each statement, indicate your agreement:
**1 = Strongly Disagree, 2 = Disagree, 3 = Neutral, 4 = Agree, 5 = Strongly Agree**
*(Higher score = more perceived overcrowding)*

| **No.** | **Statement** | **1** | **2** | **3** | **4** | **5** |
| --- | --- | --- | --- | --- | --- | --- |
| 1 | The waiting area feels crowded when I visit the hospital | ☐ | ☐ | ☐ | ☐ | ☐ |
| 2 | I often wait too long before being attended to by a doctor/nurse | ☐ | ☐ | ☐ | ☐ | ☐ |
| 3 | Hospital staff appear rushed because there are too many patients | ☐ | ☐ | ☐ | ☐ | ☐ |
| 4 | It is difficult to find a seat in the waiting area | ☐ | ☐ | ☐ | ☐ | ☐ |
| 5 | Administrative processes (registration, billing) are slowed due to overcrowding | ☐ | ☐ | ☐ | ☐ | ☐ |
| 6 | The hospital’s layout contributes to feeling congested | ☐ | ☐ | ☐ | ☐ | ☐ |
| 7 | I sometimes avoid hospital visits because of expected overcrowding | ☐ | ☐ | ☐ | ☐ | ☐ |
| 8 | Overcrowding affects the quality of care I receive | ☐ | ☐ | ☐ | ☐ | ☐ |
| 9 | The hospital lacks enough staff to handle patient load | ☐ | ☐ | ☐ | ☐ | ☐ |
| 10 | The hospital poorly manages patient flow, causing congestion | ☐ | ☐ | ☐ | ☐ | ☐ |

**Patient-Perceived Outpatient Service Quality Questionnaire (SERVQUAL – Healthcare Adapted)**

**Instructions:** For each statement, indicate your level of agreement. **1 = Strongly Disagree | 2 = Disagree | 3 = Neutral | 4 = Agree | 5 = Strongly Agree**
*(Higher scores indicate better perceived outpatient service quality)*

**A. Tangibles**

| **No.** | **Statement** | **1** | **2** | **3** | **4** | **5** |
| --- | --- | --- | --- | --- | --- | --- |
| 1 | The outpatient clinic is clean and well maintained. | ☐ | ☐ | ☐ | ☐ | ☐ |
| 2 | The waiting area has adequate seating and space. | ☐ | ☐ | ☐ | ☐ | ☐ |
| 3 | Blood pressure measuring equipment is available and functional. | ☐ | ☐ | ☐ | ☐ | ☐ |
| 4 | The clinic environment is comfortable and well ventilated. | ☐ | ☐ | ☐ | ☐ | ☐ |
| 5 | Health workers appear neat and professionally dressed. | ☐ | ☐ | ☐ | ☐ | ☐ |

**B. Reliability**

| **No.** | **Statement** | **1** | **2** | **3** | **4** | **5** |
| --- | --- | --- | --- | --- | --- | --- |
| 6 | My blood pressure is measured accurately at each visit. | ☐ | ☐ | ☐ | ☐ | ☐ |
| 7 | I receive the correct medication and prescriptions as expected. | ☐ | ☐ | ☐ | ☐ | ☐ |
| 8 | My medical records are readily available during clinic visits. | ☐ | ☐ | ☐ | ☐ | ☐ |
| 9 | Appointment schedules are followed as planned. | ☐ | ☐ | ☐ | ☐ | ☐ |
| 10 | Services provided are consistent at every clinic visit. | ☐ | ☐ | ☐ | ☐ | ☐ |

**C. Responsiveness**

| **No.** | **Statement** | **1** | **2** | **3** | **4** | **5** |
| --- | --- | --- | --- | --- | --- | --- |
| 11 | Waiting time before consultation is reasonable. | ☐ | ☐ | ☐ | ☐ | ☐ |
| 12 | Staff attend to patients promptly upon arrival. | ☐ | ☐ | ☐ | ☐ | ☐ |
| 13 | Health workers are willing to help when assistance is needed. | ☐ | ☐ | ☐ | ☐ | ☐ |
| 14 | Delays in service are clearly explained to patients. | ☐ | ☐ | ☐ | ☐ | ☐ |
| 15 | Pharmacy and laboratory services are provided without unnecessary delays. | ☐ | ☐ | ☐ | ☐ | ☐ |

**D. Assurance**

| **No.** | **Statement** | **1** | **2** | **3** | **4** | **5** |
| --- | --- | --- | --- | --- | --- | --- |
| 16 | I have confidence in the competence of healthcare providers. | ☐ | ☐ | ☐ | ☐ | ☐ |
| 17 | Health workers explain my blood pressure results clearly. | ☐ | ☐ | ☐ | ☐ | ☐ |
| 18 | Staff are polite and respectful during interactions. | ☐ | ☐ | ☐ | ☐ | ☐ |
| 19 | I feel safe receiving care at this clinic. | ☐ | ☐ | ☐ | ☐ | ☐ |
| 20 | Health workers are knowledgeable about hypertension management. | ☐ | ☐ | ☐ | ☐ | ☐ |

**E. Empathy**

| **No.** | **Statement** | **1** | **2** | **3** | **4** | **5** |
| --- | --- | --- | --- | --- | --- | --- |
| 21 | Health workers listen carefully to my concerns. | ☐ | ☐ | ☐ | ☐ | ☐ |
| 22 | I am given enough time during consultations. | ☐ | ☐ | ☐ | ☐ | ☐ |
| 23 | My privacy is respected during examinations and discussions. | ☐ | ☐ | ☐ | ☐ | ☐ |
| 24 | Health workers show concern for my personal health needs. | ☐ | ☐ | ☐ | ☐ | ☐ |
| 25 | I am treated with dignity and respect at the clinic. | ☐ | ☐ | ☐ | ☐ | ☐ |
