# Supplementary material for: Assessment of Hospital Overcrowding Perceptions and Outpatient Care Quality Among Hypertensive Patients in Mampong, Ghana: A Cross‐Sectional Study
Source: Health Sci Rep. 2026 Apr 8;9(4):e72322. doi: 10.1002/hsr2.72322 (PMC13062499; doi:10.1002/hsr2.72322)
Supplement: Supplementary file 2 — Supporting File 2: hsr272322‐sup‐0002‐Supplementary_File_2_Sensitivity_analysis [file HSR2-9-e72322-s001.docx]

**Supplementary Table 1. Linear Regression Coefficients Examining Associations Between Sociodemographic/Clinical Variables, Perceived Hospital Overcrowding, and Perceived Outpatient Care Quality**

| **Predictor Variable** | **B**  **(Unstandardized)** | **SE** | **Beta**  **(Standardized)** | **t** | **p-value** | **95% CI for B** | **Tolerance** | **VIF** |
| --- | --- | --- | --- | --- | --- | --- | --- | --- |
| Constant | 116.392 | 7.421 | — | 15.684 | <.001 | 101.794, 130.990 | — | — |
| Hospital Overcrowding | -1.491 | 0.103 | -0.641 | -14.484 | <.001 | -1.693, -1.288 | 0.639 | 1.565 |
| Age of Respondents | -2.420 | 0.651 | -0.154 | -3.720 | <.001 | -3.699, -1.140 | 0.729 | 1.373 |
| Sex | -0.717 | 1.719 | -0.016 | -0.417 | 0.677 | -4.099, 2.664 | 0.842 | 1.188 |
| Marital Status | 0.478 | 0.879 | 0.023 | 0.544 | 0.587 | -1.251, 2.206 | 0.720 | 1.388 |
| Educational Level | 1.914 | 0.847 | 0.096 | 2.261 | 0.024 | 0.249, 3.580 | 0.698 | 1.433 |
| Monthly Income (GHS) | 1.933 | 0.819 | 0.101 | 2.362 | 0.019 | 0.323, 3.543 | 0.691 | 1.446 |
| Place of Residence | -0.081 | 1.145 | -0.003 | -0.071 | 0.944 | -2.334, 2.172 | 0.563 | 1.776 |
| Insurance | 0.929 | 1.614 | 0.021 | 0.576 | 0.565 | -2.246, 4.104 | 0.929 | 1.076 |
| Duration of Illness | -0.200 | 1.194 | -0.006 | -0.167 | 0.867 | -2.549, 2.150 | 0.956 | 1.046 |
| Comorbidities | -2.622 | 2.233 | -0.060 | -1.174 | 0.241 | -7.014. 1.770 | 0.481 | 2.077 |

**Notes:** Dependent variable: Perceived Outpatient Service Quality (continuous), SE = Standard Error; CI = Confidence Interval; VIF = Variance Inflation Factor. This table serves as supplementary material for sensitivity analysis using linear regression to confirm the direction of associations observed in the main logistic regression.
